# The complex diagnosis of post-dialysis fever: a case report and literature review of infective endocarditis in a dialysis patient

**DOI:** 10.1186/s12882-025-04236-7

**Published:** 2025-07-01

**Authors:** Xinyu Wang, Yu Xie, Meiyu Chen, Hongyan Zhu, Guonian He, Wenjing Yu, Dan Qiao, Ying Shen, Lu Song, Qinyuan Deng

**Affiliations:** 1 Department of Nephrology, The First People’s Hospital of Yunnan Province, 157 Jinbi Road, Kunming, Yunnan 650032 China; 2 The Affiliated Hospital of Kunming University of Science and Technology, 157 Jinbi Road, Kunming, Yunnan 650032 China; 3 Department of Clinical Laboratory, The First People’s Hospital of Yunnan Province, 157 Jinbi Road, Kunming, Yunnan 650032 China; 4 Department of Integrated Traditional Chinese and Western Medicine, The First People’s Hospital of Yunnan Province, 157 Jinbi Road, Kunming, Yunnan 650032 China; 5 Department of Infection I, The Third People’s Hospital of Kunming, Kunming, Yunnan China

**Keywords:** Post-dialysis fever, Infective endocarditis (IE), Transesophageal echocardiography (TEE), Next-generation sequencing (NGS)

## Abstract

**Background:**

Post-dialysis fever is a common but diagnostically challenging issue in hemodialysis patients, with potential causes including dialysis-related infections, pulmonary infections, and cardiovascular complications.

**Case presentation:**

We report a 76-year-old male with end-stage renal disease (ESRD) on maintenance hemodialysis, coronary artery disease, and prior cardiac stent implantation, who presented with recurrent post-dialysis fever. Despite persistently negative conventional cultures, metagenomic next-generation sequencing (NGS) of pre-dialysis blood samples identified *Pseudomonas aeruginosa* (*P. aeruginosa*), *Cutibacterium acnes* (*C. acnes;* formerly *Propionibacterium acnes*), *Staphylococcus epidermidis* (*S. epidermidis*), *and Corynebacterium accolens* (*C. accolens*) and Epstein-Barr virus (EBV), while post-dialysis samples revealed only *C. acnes* and EBV. Given the temporal association with fever, these two pathogens were considered the primary causative agents. Subsequent transesophageal echocardiography (TEE) confirmed aortic valve vegetations, establishing the diagnosis of infective endocarditis (IE). Following targeted antimicrobial and antiviral adjustments based on NGS findings, the patient exhibited complete resolution of post-dialysis fever and was discharged. However, as the vegetation was not surgically removed, he was hospitalized multiple times over the following five months for recurrent infections and ultimately died of septic shock and multi-organ failure due to carbapenem-resistant *Klebsiella pneumoniae*.

**Conclusions:**

This case underscores the complementary role of TEE and NGS in diagnosing IE in high-risk patients, enabling the detection of uncommon pathogens and informing targeted therapy to improve clinical outcomes.

**Clinical trial number:**

Not applicable.

**Supplementary Information:**

The online version contains supplementary material available at 10.1186/s12882-025-04236-7.

## Introduction

Post-dialysis fever is a frequent but diagnostically challenging clinical presentation in hemodialysis patients, with causes ranging from dialysis-related infections to pulmonary or systemic infectious diseases [1–3]. Research shows that patients with end-stage renal disease (ESRD) are at significantly higher risk for infective endocarditis (IE), with incidence rates 50–60 times greater than those of the general population. Age-adjusted incidence and relative risk ratios reach 17.86 and 16.90, respectively [4–6]. Among patients with coronary artery disease and cardiac stent implantation, IE must be considered in differential diagnoses, as its clinical manifestations are often atypical, leading to misdiagnosis or delayed diagnosis [7, 8]. This case explores the diagnostic utility of next-generation sequencing (NGS) and transesophageal echocardiography (TEE) in identifying IE in complex clinical scenarios.

## Case presentation

### Clinical history

A 76-year-old male with ESRD on maintenance hemodialysis for three years (3 sessions per week, 4 h per session) via a left forearm arteriovenous fistula (AVF) presented with a two-week history of intermittent fever, cough, and sputum production. His medical history included 20 years of hypertension managed with amlodipine and sacubitril/valsartan, 20 years of diabetes treated with insulin, and coronary artery disease treated with stent implantation in December 2023. The patient’s surgical history includes the creation of a left forearm AVF in July 2021 for maintenance hemodialysis, surgical resection of a bladder tumor in October 2021, left knee arthroplasty in June 2021, and cervical abscess drainage in December 2022. Postoperative pathology of the cervical abscess showed chronic granulomatous inflammation with suppurative changes; cultures were negative.

### First hospitalization

**Fig. 1:**
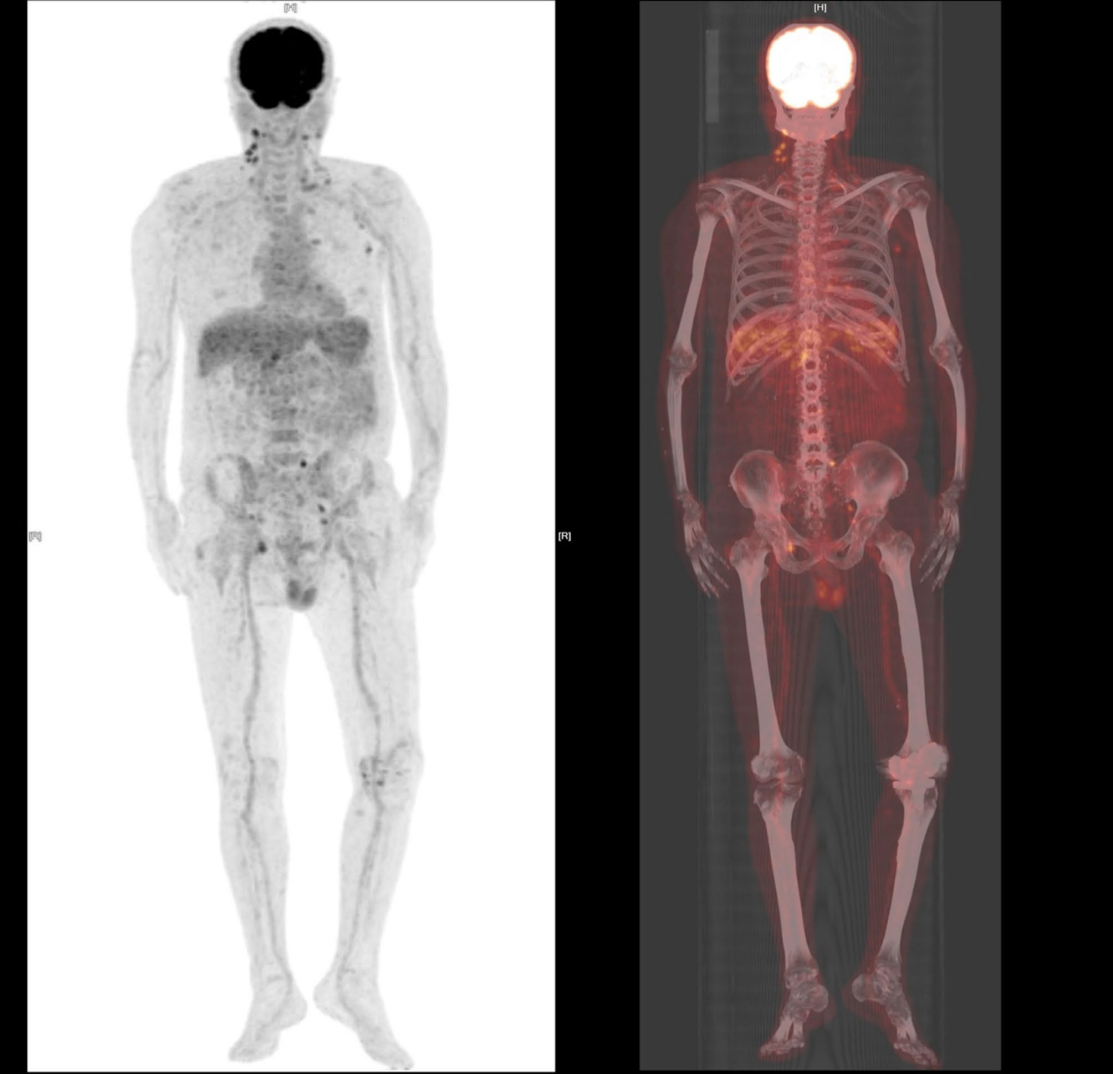
PET/CT Imaging of Whole-Body. (1) Postoperative bladder malignancy: The bladder shows reduced volume with low metabolic activity, with no definitive signs of tumor recurrence; (2) Multiple lymph nodes in the hepatic-gastric space, porta hepatis, bilateral inguinal regions, bilateral carotid sheath, clavicular, and bilateral axillary areas: Some of these lymph nodes are slightly enlarged with mild to moderate metabolic increase, which is more consistent with reactive lymphadenopathy; (3) Bilateral renal atrophy with faint imaging findings; (4) Age-related cerebral changes; (5) Multiple chronic inflammatory and calcified foci in both lungs, with small amounts of pleural effusion on both sides; (6) Multiple calcific lesions observed in the aortic valve, mitral valve, bilateral coronary arteries, thoracoabdominal aorta, and some branches; (7) Post bilateral knee joint replacement: Mildly increased metabolic activity in the synovial membranes; increased metabolism at the attachment site of the right ischial tuberosity and obturator internus muscle, suggesting inflammatory uptake

On October 25, 2024, upon admission, physical examination revealed only mild cervical lymphadenopathy. Notably, a prior cervical lymph node biopsy had demonstrated chronic granulomatous inflammation with suppurative changes. A soft precordial murmur was auscultated, while the remainder of the systemic examination was unremarkable. Blood cultures remained consistently negative before and after febrile episodes, dialysis procedures, and empirical antibiotic therapy, while sputum culture identified *Acinetobacter urinae* and microscopy revealed yeast-like fungal spores. Laboratory data (Table 1) showed a markedly elevated inflammatory response, with an erythrocyte sedimentation rate (ESR) of 83 mm/h, high-sensitivity C-reactive protein (hsCRP) > 200 mg/L, and procalcitonin (PCT) at 28.89 pg/mL. The white blood cell (WBC) count was within normal range, but mild lymphopenia was observed. Notably, cardiac biomarkers were elevated, with N-terminal pro B-type natriuretic peptide (NT-proBNP) reaching 6324 pg/mL, suggesting underlying cardiac strain. Imaging, including abdominal and vascular ultrasound, chest CT, transthoracic echocardiography (TTE) and whole-body PET/CT (Fig. 1), revealed no clear source of infection other than chronic inflammatory changes in the lungs and bilateral knee. Initial empirical therapy with piperacillin-tazobactam (4.5 g IV every 8 h) for 6 days failed to resolve post-dialysis fever. In response, antimicrobial therapy was escalated to meropenem (0.5 g IV every 8 h), which effectively controlled the fever, and no recurrence was noted prior to discharge on hospital day 18.

**Table 1:** Laboratory findings

Project	First hospitalization	Second hospitalization
ESR	83 mm/h	86 mm/h
hsCRP	> 200 mg/L	183.41 mg/L
PCT	28.89 pg/mL	57.03 ng/mL
WBC	9.62 × 10^9^/L	3.75 × 10^9^/L
NEUT	7.48 × 10^9^/L	2.76 × 10^9^/L
LYMPH	1.30 × 10^9^/L	0.71 × 10^9^/L
MONO	0.63 × 10^9^/L	0.20 × 10^9^/L
RBC	4.13 × 10^12^/L	3.72 × 10^12^/L
PLT	227 × 10^12^/L	80 × 10^12^/L
HB	112 g/L	101 g/L
AST	15 U/L	55 U/L
ALT	13 U/L	21 U/L
TBIL	10.4 µmol/L	10.6 µmol/L
DBIL	6.2 µmol/L	4.7 µmol/L
UBIL	4.2 µmol/L	5.9 µmol/L
TP	75.5 g/L	76.3 g/L
ALB	36.9 g/L	34.2 g/L
Cr	765 µmol/L	1027 µmol/L
NT-proBNP	6324 pg/mL	16,954 pg/mL
CK-MB	1.6 ng/mL	0.3 ng/mL
MYO	614.4 ng/mL	342.5 ng/mL
aTnI	0.04 ng/mL	0.027 ng/mL
IgG	18.7 g/L	17.10 g/L
IgA	5.17 g/L	8.82 g/L
KAP	4.83 g/L	6.03 g/L
LAM	2.36 g/L	3.27 g/L
CD3 + CD4+	N/A	58.95%
CD4/CD8	N/A	3.27
Sputum culture (Fever/No fever)	*Acinetobacter urinae*, yeast-like fungal spores	Negative
Blood culture (Fever/No fever)	Negative	Negative

Erythrocyte Sedimentation Rate (ESR), High-Sensitivity C-Reactive Protein (hsCRP), Procalcitonin (PCT), white blood cell count (WBC), Neutrophils (NEUT), Lymphocytes (LYMPH), Monocytes (MONO), Hemoglobin (HB), Platelets (PLT), Aspartate Aminotransferase (AST), Alanine Aminotransferase (ALT), Total Bilirubin (TBIL), Direct Bilirubin (DBIL), Unconjugated Bilirubin (UBIL), Total Protein (TP), Albumin (ALB), Creatinine (Cr), Glomerular Filtration Rate (GFR-EPI), N-Terminal Pro B-Type Natriuretic Peptide (NT-proBNP), Creatine Kinase-MB (CK-MB), Myoglobin (MYO), Cardiac Troponin I (aTnI), Immunoglobulin G (IgG), Immunoglobulin A (IgA), Kappa Light Chains (KAP), Lambda Light Chains (LAM), CD3 + CD4 + T Lymphocytes (CD3 + CD4+), and CD4/CD8 T-Cell Ratio

### Second hospitalization

**Fig. 2:**
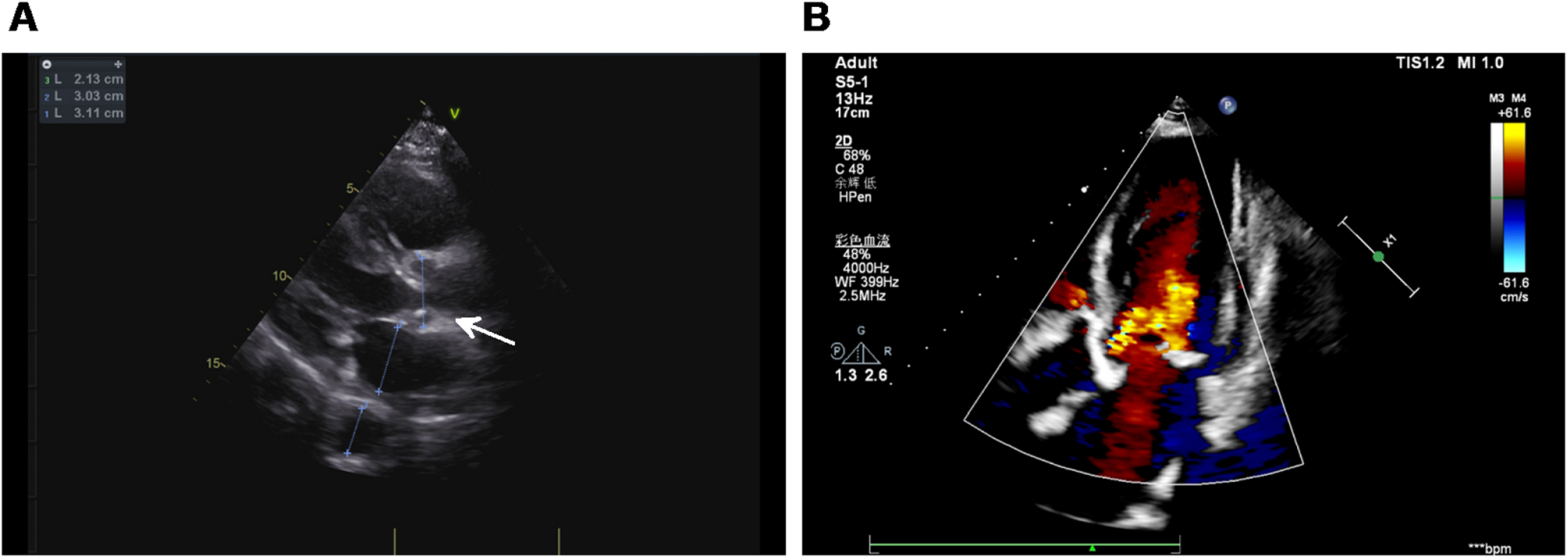
Echocardiographic assessment of aortic valve function. (**A**) Two-dimensional echocardiography reveals mild aortic valve stenosis and incomplete valve closure (white arrow). (**B**) Color Doppler imaging demonstrates turbulent flow consistent with mild-to-moderate aortic stenosis

On November 20, the patient was readmitted due to recurrent post-dialysis fever, cough, and sputum production. Repeat TTE showed mild aortic valve stenosis and regurgitation (Fig. 2). Inflammatory markers remained significantly elevated, with ESR at 86 mm/h, hsCRP at 183.41 mg/L, and PCT increased to 57.03 ng/mL. A decline in WBC count (3.75 × 10⁹/L) was accompanied by progressive neutropenia and lymphopenia. NT-proBNP rose sharply to 16,954 pg/mL, indicating worsening cardiac dysfunction, which further supported the suspicion of IE in the absence of other clear infectious sources. Repeated blood and sputum cultures consistently yielded negative results. A comprehensive infectious workup was performed, including evaluation for respiratory pathogens, acid-fast bacilli, and sputum culture analysis, all of which yielded unremarkable findings. Notably, serological analysis of Epstein-Barr virus (EBV) specific antibodies revealed positive IgG responses against both viral capsid antigen (VCA) and nuclear antigen (EBNA), while other EBV-related antibodies remained negative. This serological profile is consistent with a past EBV infection, indicating prior exposure to the virus. Despite treatment with meropenem (0.5 g IV every 8 h), symptoms persisted.

**Fig. 3:**
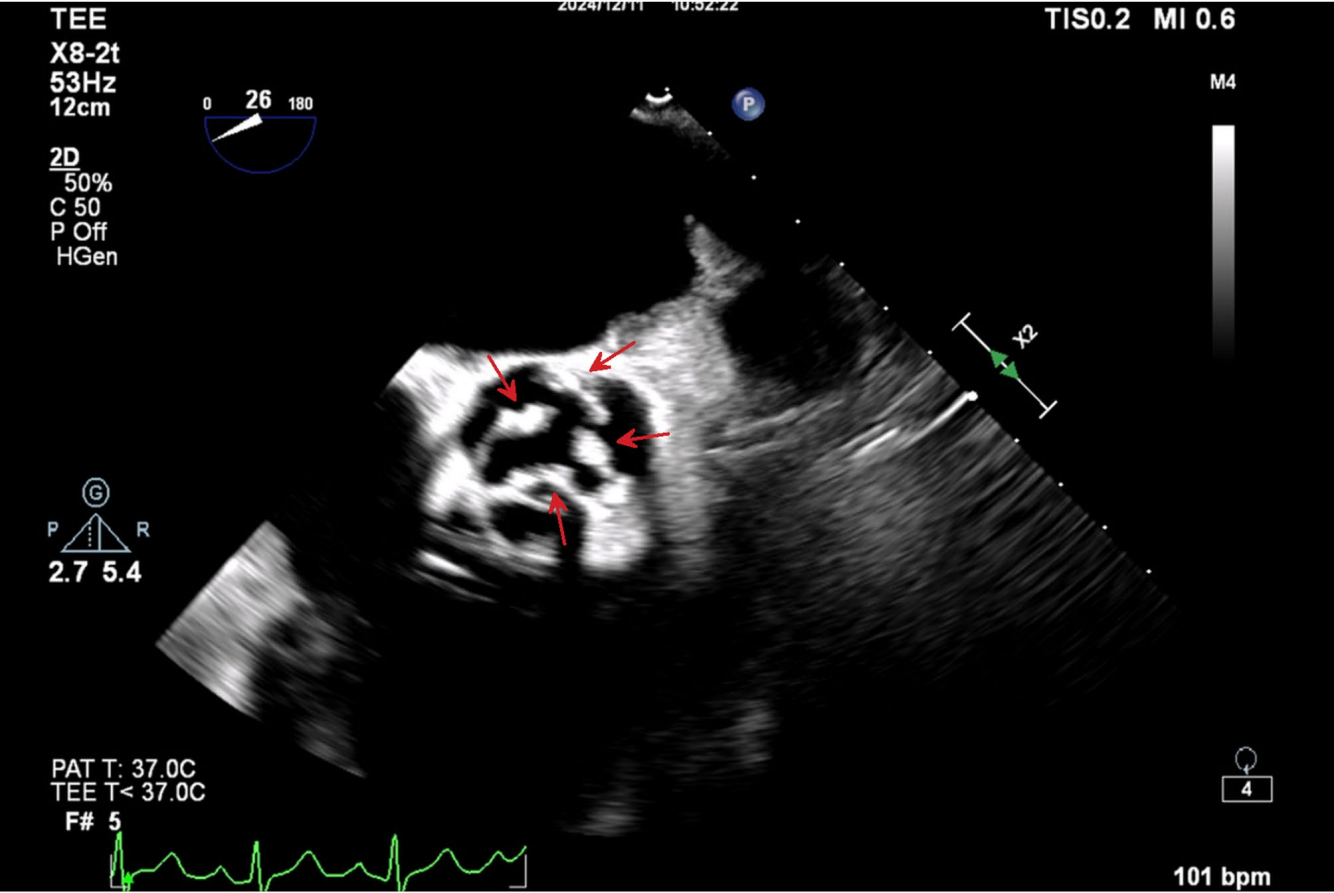
Transesophageal echocardiogram (TEE) showing aortic valve vegetation. TEE reveals multiple irregular, high-density vegetations attached to the aortic valve and annular root (red arrows), with measured sizes of 0.29 × 0.26 cm, 0.51 × 0.35 cm, 0.81 × 0.25 cm, and 0.54 × 0.32 cm

**Fig. 4:**
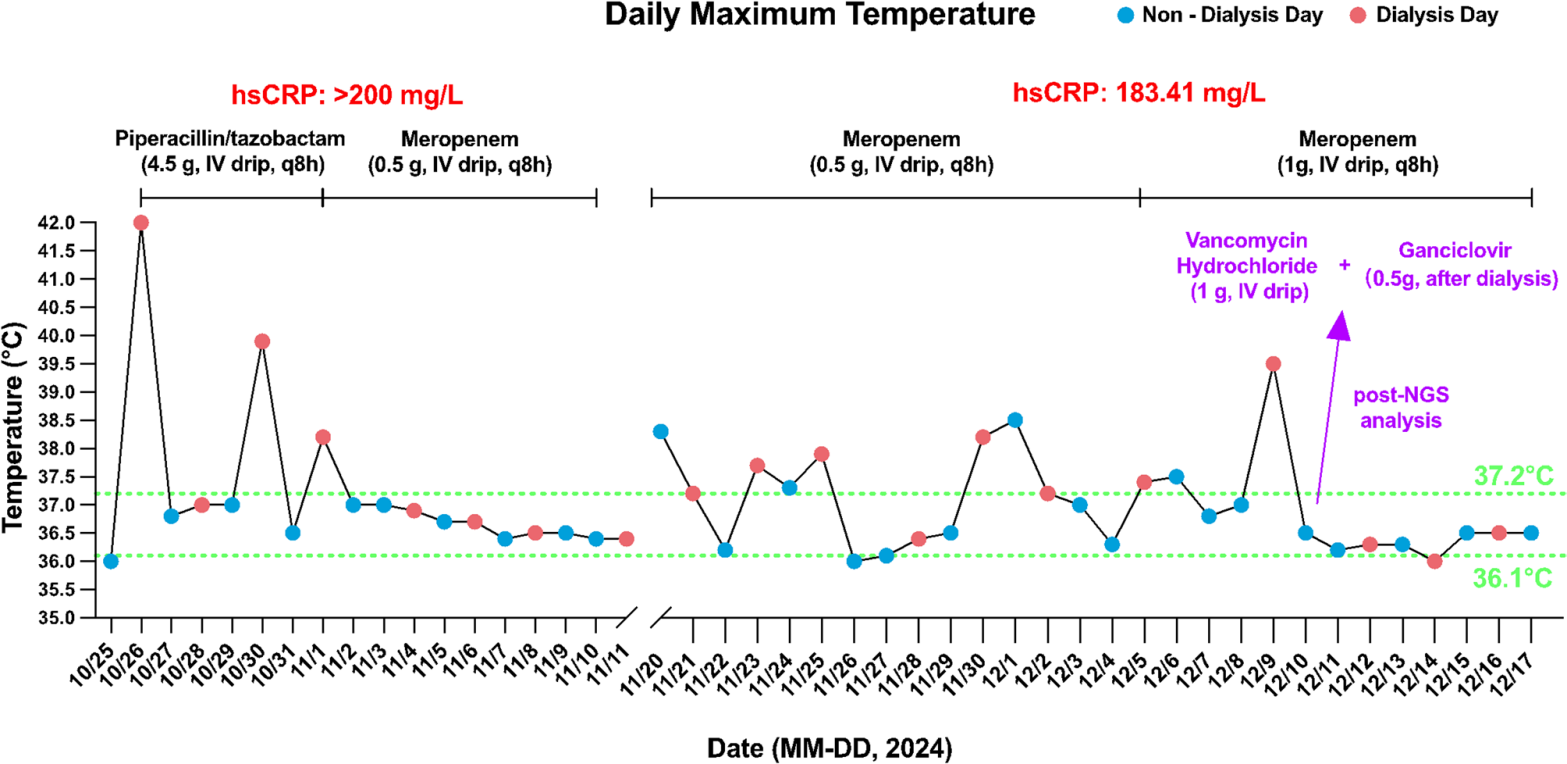
Daily maximum temperature trends and medication administration. High-Sensitivity C-Reactive Protein (hsCRP), Intravenous (IV), Next-generation sequencing (NGS), Every 8 h (q8h)

NGS of pre-dialysis blood samples detected *Pseudomonas aeruginosa* (*P. aeruginosa*, 12 reads, 17.5% relative abundance) and EBV DNA (4 reads). Other identified taxa included *Cutibacterium acnes* (*C. acnes*, 28 reads, 37.5%), *Staphylococcus epidermidis* (*S. epidermidis*, 8 reads, 12.5%), and *Corynebacterium accolens* (*C. accolens*, 8 reads, 3%). However, these three bacterial species recognized as common human commensal microbiota, were excluded from the initial clinical report due to their ubiquitous colonization in healthy populations and limited diagnostic relevance. Post-dialysis NGS analysis revealed a marked shift in microbial composition, with *C. acnes* predominating (6 reads, 53.8% relative abundance). EBV DNA remained detectable at reduced levels (2 reads). Despite its commensal nature, the substantial increase in *C. acnes* relative abundance (exceeding 50% post-dialysis) warranted inclusion in the clinical report. This observation may reflect altered host-microbiota dynamics or potential opportunistic infection associated with dialysis-related immune modulation. Antibiotics were adjusted to vancomycin (1 g IV every 8 h) and oral ganciclovir (0.5 g after each hemodialysis session), with meropenem (1 g IV every 8 h). The patient’s temperature gradually returned to normal, and no recurrence of fever was observed prior to discharge. Based on the consistent pattern of post-dialysis fever, it was hypothesized that the patient’s symptoms were likely due to hemodynamic changes induced by dialysis, which in turn led to transient bacteremia. Considering the patient’s history of stent implantation, a TEE was further conducted, revealing a vegetation on the aortic valve **(**Fig. 3**)**. The vegetation appeared as a mass with band-like high-density proliferative features, with a maximum diameter of 0.81 × 0.25 cm. The patient and his family declined surgical removal of vegetations, and he was discharged on December 17. A timeline of clinical events is provided in Fig. 4.

### Follow-up

After discharge, the patient was rehospitalized multiple times at Third People’s Hospital of Kunming (Yunnan Provincial Clinical Center for Infectious Diseases) due to post-dialysis fever. Although IE was repeatedly controlled with antibiotics, the patient ultimately died from septic shock and multiple organ failure secondary to pulmonary infection caused by carbapenem-resistant *Klebsiella pneumoniae* on April 21, 2025. Detailed clinical course is provided in the Supplementary Materials.

## Discussion

This case describes a 76-year-old male with ESRD on maintenance hemodialysis who presented with recurrent post-dialysis fever. Despite negative blood cultures and EBV serology suggesting past infection, NGS identified *P. aeruginosa*, *C. acnes*, *S. epidermidis*, *C. accolens*, and EBV DNA in pre-dialysis blood samples, while only *C. acnes* and EBV were detected post-dialysis. Given the consistent onset of fever after dialysis sessions, *C. acnes* and EBV were considered the primary causative agents. TEE confirmed aortic valve vegetation, establishing the diagnosis of infective endocarditis. The patient improved with adjusted antibiotic therapy but declined surgery. He later died from septic shock and multi-organ failure due to carbapenem-resistant *Klebsiella pneumoniae* infection.

Due to the small size of the vegetation in this case, TTE revealed only mild aortic valve stenosis and regurgitation. Notably, repeated TTE examinations over the subsequent five months continued to show no visible vegetations, further highlighting the limitations of TTE in such contexts. In contrast, TEE clearly identified the aortic valve vegetation. This underscores the importance of prioritizing high-sensitivity imaging modalities, such as TEE, in the diagnostic evaluation of complex infectious diseases. Literature reports indicate that the size of the vegetation significantly affects the diagnostic sensitivity of TTE, with only 25% of vegetations smaller than 5 mm and 70% of those measuring 6–10 mm being detectable. In addition, underlying valvular diseases, such as myxomatous degeneration of the mitral valve or calcification of the valve, can further reduce the accuracy of TTE. In contrast, TEE, with its higher resolution and multi-angle imaging capabilities, has significantly better diagnostic sensitivity, reaching 90-100%, while TTE’s sensitivity is only 40-63% [9]. Therefore, TEE should be considered a routine diagnostic tool for IE in high-risk populations like hemodialysis patients, who have unique hemodynamic changes.

Fever in hemodialysis patients is most commonly attributed to catheter-related infections, contamination of dialysis water, or equipment-associated infections [10, 11]. In our unit, all aspects of dialysis practice—including water treatment, environmental control, and procedural operations—were conducted in strict accordance with the national guideline, *Blood Purification Standard Operating Procedure (SOP) – 2021 Edition* [in Chinese]. As the patient showed no improvement after replacement of the dialysis machine and hemofilter, and no similar febrile episodes occurred in other patients, these potential sources of infection were considered unlikely. Given that hemodialysis patients are at significantly higher risk for IE due to immunosuppression, repeated vascular access interventions, and underlying comorbidities, the possibility of IE was carefully considered. This is particularly relevant in patients with coronary artery disease or a history of cardiac stent implantation. A study based on the 2006–2011 Nationwide Inpatient Sample (NIS) reported that approximately 2-5% of ESRD dialysis patients develop IE, with *Staphylococcus aureus* being the most common pathogen, accounting for 61% of cases, while Gram-negative bacteria, streptococci, and enterococci are less common [12]. NGS, an emerging molecular diagnostic technique, offers significant advantages in pathogen identification, especially in complex cases with negative routine cultures [13–17].

In this case, NGS revealed the presence of *C. acnes* and EBV, all of which are uncommon pathogens in IE. However, given the patient’s long-term dialysis treatment leading to immunosuppression, these pathogens are of significant clinical relevance. Although EBV infection is rarely reported in IE, it can lead to severe cardiovascular complications, including coronary artery aneurysms, coronary endocarditis, myocarditis, and heart failure [18]. Previous reports have shown that EBV-related valve disease generally has a relatively good prognosis, with the severity of regurgitation depending on the degree of valve damage [19, 20]. Nevertheless, emerging evidence indicates that EBV may also cause serious myocardial injury, even in immunocompetent individuals. Reported cases include EBV-associated myopericarditis, fatal myocarditis in chronic active EBV infection, and sudden cardiac death with EBV DNA detected in myocardial tissue [21–23]. These findings highlight the potential for EBV to induce severe cardiac pathology, even in the absence of classic clinical signs. In our case, serology indicated prior EBV exposure (positive VCA-IgG and EBNA-IgG), but low-level EBV DNA was detected in both pre- and post-dialysis blood by NGS, suggesting possible subclinical reactivation under systemic stress. Given the exclusion of common pathogens and persistent fever, EBV was considered a potential contributor. Despite the uncertain pathogenic significance of EBV, the detection of EBV DNA by NGS underscores the need to consider viral reactivation in dialysis patients with unexplained fever and systemic inflammation.

Similarly, *P. aeruginosa*-related IE is rare and has a poor prognosis. It is more commonly associated with right-sided heart infections in intravenous drug users or with prosthetic valves and cardiac devices, often requiring early combination antibiotic therapy and surgical intervention [24, 25]. *Cutibacterium* species, while rare causes of IE, pose diagnostic challenges due to their slow growth and the frequent misinterpretation of positive blood cultures as contamination from skin flora. A Swedish study of IE cases from 1995 to 2016 found that *Cutibacterium* species caused IE in nearly all male patients with prosthetic heart valves, accounting for 8% of prosthetic valve IE cases. The study emphasized the importance of extending blood culture incubation time to 14 days and noted that most patients required surgical intervention, though the cure rate remained as high as 92.1%, indicating a favorable prognosis [26]. *Corynebacterium Species* and *S. epidermidis* are part of the normal human microbiota but have also been reported as causative agents of IE, particularly in patients with prosthetic heart valves [27, 28]. Due to their commensal nature, these organisms are often overlooked in clinical settings, which may lead to delayed diagnosis and treatment. In our case, although *P. aeruginosa*, *C. accolens* and *S. epidermidis* were also detected in pre-dialysis samples, only *C. acnes* remained detectable and increased in the post-dialysis blood specimens, while EBV DNA levels decreased. Given the temporal correlation between hemodialysis sessions and fever onset, we hypothesize that *C. acnes* and EBV were the primary causative agents of IE in this case. This conclusion guided our therapeutic strategy, leading to the addition of vancomycin and ganciclovir to the treatment regimen, which resulted in favorable clinical outcomes. Although ganciclovir is primarily indicated for cytomegalovirus (CMV) infections, its known inhibitory effect on EBV replication supported its empirical use in this case [29, 30].

The mortality of IE is often related to severe complications involving the heart, central nervous system, lungs, or other organs. Literature reports indicate that 57% of IE patients develop at least one complication, 26% develop two, and 14% have three or more [31]. In our case, the patient experienced multiple complications, including recurrent fever, seizures, altered consciousness, hypotensive shock, respiratory failure, and bone marrow suppression. Although echocardiography did not reveal a periannular abscess, persistent inflammation, neurological symptoms, and limited antibiotic response suggested advanced disease progression beyond valvular structures. According to the guidelines of the European Society of Cardiology (ESC) and the American Heart Association (AHA), surgical indications for IE include acute heart failure, uncontrolled infection (such as periannular abscess or persistent bacteremia lasting ≥ 7 days), and large vegetations (> 10–15 mm) associated with high embolic risk [32, 33]. In our case, TEE revealed an aortic valve vegetation measuring 0.81 × 0.25 cm, which did not meet the guideline-defined threshold for surgical intervention. Following multidisciplinary consultation, and considering the patient’s initial response to antibiotic therapy, advanced age, multiple comorbidities, and limited surgical tolerance, both the patient and his family elected to forego surgery in favor of conservative medical management. Notably, a randomized trial showed that early surgery in IE patients with large vegetations significantly reduced the 6-week composite endpoint of death and embolism (3% vs. 23%, HR 0.10, *P* = 0.03) and improved 6-month outcomes [34]. Although our patient did not meet surgical criteria at the time, the later neurologic and infectious deterioration suggests that earlier intervention might have been beneficial.

We hypothesize that transient bacteremia following dialysis, primarily caused by *C. acnes* and EBV colonizing the endocardium, was the underlying trigger of post-dialysis fever. However, the findings may have been affected by technical factors such as potential contamination with skin flora during sample collection and amplification bias inherent to 16 S rRNA sequencing. The timing of NGS testing in relation to antimicrobial therapy also presents a potential confounder. In this case, NGS was performed after initiation of empirical antibiotic treatment, which may have partially suppressed susceptible organisms and skewed the microbial profile toward more resilient or low-virulence species such as *C. acnes*. Therefore, while our hypothesis is biologically plausible and supported by the temporal association between fever onset and microbial changes, it remains speculative and should be interpreted with caution. Additionally, the absence of surgical removal of valvular vegetations limited our ability to obtain pathological confirmation of the causative organism. As summarized in Table 2, our findings highlight the potential utility of NGS in uncovering atypical or polymicrobial etiologies of infective endocarditis, particularly in hemodialysis patients with diagnostically challenging presentations. Further case accumulation and validation in larger cohorts are warranted to develop standardized interpretive criteria for the clinical application of NGS in IE.

**Table 2:** NGS-driven pathogen identification in culture-negative IE: case-literature comparative analysis

Blood culture	Treatment	Days interval	NGS	Treatment after NGS	Outcome	Reference
Negative	Piperacillin/tazobactam, Meropenem	6 days	*Cutibacterium acnes* and Epstein-Barr virus	Meropenem, Vancomycin and Ganciclovir	Transient improvement; death at 5 months	The present study
Negative	Moxifloxacin	10 days	*Coxiella burnetii*	Minocycline	Improvement	2024 [35]
Negative	Imipenem and Teicoplanin	Above 10 days	*Mycobacterium kansasii*	Rifampicin, Ethambutol, Azithromycin and Isoniazid	Death	2023 [36]
Negative	Vancomycin and Ceftriaxone	10 days	*Coxiella burnetii*	Doxycycline	Improvement	2022 [37]
Negative	Vancomycin, Ceftriaxone and Azithromycin	5 days	*Bartonella henselae*	Doxycycline and Rifampin (After renal biopsy, gentamicin was substituted for rifampin)	Death	2022 [38]
Negative	Meropenem and Vancomycin	9 days	*Aspergillus flavus*	Voriconazole and moxifloxacin	Improvement	2021 [13]
Negative	Amoxicillin, Amoxicillin/clavulanic acid and Gentamicin	7 days	*Cryptosporidium hominis*	Ceftriaxone	Improvement	2019 [39]

Next-generation sequencing (NGS); Days interval: The interval between the last negative culture and NGS testing

In conclusion, this case highlights the critical role of advanced imaging and molecular diagnostic technologies in managing IE, optimizing diagnostic workflows, enhancing treatment outcomes, and exploring the mechanisms and management strategies for co-infections by complex pathogens.

## Electronic supplementary material

Below is the link to the electronic supplementary material.

Supplementary Material 1

## Data Availability

The datasets generated and/or analysed during the current study are available in the NCBI repository, [https://www.ncbi.nlm.nih.gov/bioproject/PRJNA1228522].
