# Supplementary material for: The complex diagnosis of post-dialysis fever: a case report and literature review of infective endocarditis in a dialysis patient
Source: BMC Nephrol. 2025 Jul 1;26:331. doi: 10.1186/s12882-025-04236-7 (PMC12219980; doi:10.1186/s12882-025-04236-7)
Supplement: Supplementary file 1 — Supplementary Material 1 [file 12882_2025_4236_MOESM1_ESM.docx]

**Follow-up**

On January 14, 2025, the patient was hospitalized again at Third People's Hospital of Kunming City due to post-dialysis fever. Laboratory tests revealed WBC 10.10 × 10⁹/L, NEUT 7.12 × 10⁹/L, LYMPH 1.48 × 10⁹/L, MONO 1.06 × 10⁹/L, RBC 2.87 × 10¹²/L, and Hb 78 g/L, indicating mild leukocytosis and normocytic anemia. Inflammatory markers were significantly elevated, with hsCRP at 149.61 mg/L, IL-6 at 31.14 pg/mL, and PCT at 16.84 ng/mL. Initial blood and sputum cultures were negative. The patient was empirically started on cefoperazone-sulbactam (1 g IV every 24 hours), but his fever persisted without clinical improvement. TTE performed on January 16 showed mild aortic valve stenosis and regurgitation, mitral valve calcification with mild reflux, and no visible vegetation. On January 24, sputum culture identified *Staphylococcus haemolyticus*, leading to a switch in antimicrobial regimen to imipenem/cilastatin (0.5 g IV every 6 hours) combined with linezolid (0.6 g IV every 12 hours). The patient's symptoms improved and he was discharged.

On February 11, he was readmitted following four episodes of seizures occurring immediately before or after dialysis sessions. Blood and sputum cultures remained negative throughout hospitalization. Empirical treatment with ertapenem (0.3 g IV every 8 hours) and linezolid (0.6 g IV every 12 hours) was initiated. The seizures did not recur, and the patient was discharged on February 27 in stable condition.

On March 13, the patient was again admitted with recurrent fever, a new-onset seizure, and transient loss of consciousness. Laboratory evaluation showed WBC 2.89 × 10⁹/L, NEUT 1.74 × 10⁹/L, LYMPH 0.55 × 10⁹/L, MONO 0.52 × 10⁹/L, RBC 1.91 × 10¹²/L, and Hb 57 g/L, suggesting worsening anemia and mild leukopenia. Inflammatory markers remained elevated (hsCRP 38.42 mg/L, IL-6 16.25 pg/mL, and PCT 1.81 ng/mL). The patient received cefoperazone-sulbactam (1 g IV every 24 hours) and oral linezolid (0.6 g every 12 hours), which resulted in transient clinical improvement. Given the absence of new findings on imaging and stabilized vitals, IE was considered clinically controlled, and intravenous antibiotics were tapered beginning on March 17, with oral linezolid continued as suppressive therapy.

However, on March 20, the patient experienced a sudden deterioration characterized by high fever, generalized convulsions, hypotension (BP 71/41 mmHg), and tachycardia (HR 117 bpm). Emergency management included endotracheal intubation and continuous norepinephrine infusion at a total dose of 10 mg, administered via micropump at 1 mL/h. Repeat TTE again showed mild aortic stenosis and regurgitation, along with mitral valve calcification and moderate reflux, but no visible vegetation. Chest CT demonstrated diffuse miliary nodules, patchy and streaky pulmonary infiltrates, and mild pleural effusion.

On March 24, sputum culture yielded carbapenem-resistant *Klebsiella pneumoniae*. The antibiotic regimen was adjusted to biapenem (0.3 g IV every 24 hours) and linezolid (0.6 g IV every 12 hours). However, inflammatory markers remained elevated (hsCRP 49.30 mg/L, IL-6 27.69 pg/mL, and PCT 4.31 ng/mL on March 26), indicating a poor response. Tigecycline (50 mg IV every 12 hours) was subsequently added. By April 9, sputum nucleic acid testing detected *Candida albicans*, *Candida tropicalis*, and *Candida glabrata*. The serum (1,3)-β-D-glucan level was 148.18 pg/mL, consistent with systemic fungal infection. Caspofungin (50 mg IV every 24 hours) was initiated as antifungal therapy. On April 13, brain CT showed symmetrical bilateral cerebral hemispheres with clear gray-white matter differentiation. The bilateral lateral ventricles and third ventricle were enlarged, without evidence of compression or deformation of the ventricular system. The cerebellum and brainstem appeared normal in morphology.

Despite broad-spectrum antimicrobial and antifungal coverage, follow-up chest CT showed progression of pleural effusion with localized pleural thickening. On April 20–21, laboratory evaluation revealed profound myelosuppression and severe anemia: WBC 2.62 × 10⁹/L, NEUT 2.16 × 10⁹/L, LYMPH 0.37 × 10⁹/L, MONO 0.09 × 10⁹/L, RBC 1.09 × 10¹²/L, and Hb 33 g/L. The patient ultimately succumbed to septic shock and multi-organ failure on April 21.

Surgical removal of the aortic valve vegetation had been discussed during the initial hospitalization. However, due to the patient's frailty and limited surgical tolerance, the family opted for conservative medical management. After the patient’s death, pathological examination of the valve tissue was suggested but declined by the family.
